# Cost-Effectiveness of HIV Drug Resistance Testing to Inform Switching to Second Line Antiretroviral Therapy in Low Income Settings

**DOI:** 10.1371/journal.pone.0109148

**Published:** 2014-10-07

**Authors:** Andrew Phillips, Valentina Cambiano, Fumiyo Nakagawa, Travor Magubu, Alec Miners, Debbie Ford, Deenan Pillay, Andrea De Luca, Jens Lundgren, Paul Revill

**Affiliations:** 1 Research Department of Infection & Population Health, UCL, London, United Kingdom; 2 University of Zimbabwe Clinical Research Centre, Harare, Zimbabwe; 3 Department of Health Services Research and Policy, London School of Hygiene and Tropical Medicine, London, United Kingdom; 4 MRC Clinical Trials Unit, UCL, London, United Kingdom; 5 Africa Centre, KwaZulu Natal, South Africa; 6 University Division of Infectious Diseases, Siena University Hospital, Siena, Italy; 7 Dept of Infectious Diseases, Rigshospitalet, University of Copenhagen, Copenhagen, Denmark; 8 Centre for Health Economics, University of York, York, United Kingdom; Johns Hopkins Bloomberg School of Public Health, United States of America

## Abstract

**Background:**

To guide future need for cheap resistance tests for use in low income settings, we assessed cost-effectiveness of drug resistance testing as part of monitoring of people on first line ART - with switching from first to second line ART being conditional on NNRTI drug resistance mutations being identified.

**Methods:**

An individual level simulation model of HIV transmission, progression and the effect of ART which accounts for adherence and resistance development was used to compare outcomes of various potential monitoring strategies in a typical low income setting in sub-Saharan Africa. Underlying monitoring strategies considered were based on clinical disease, CD4 count or viral load. Within each we considered a strategy in which no further measures are performed, one with a viral load measure to confirm failure, and one with both a viral load measure and a resistance test. Predicted outcomes were assessed over 2015–2025 in terms of viral suppression, first line failure, switching to second line regimen, death, HIV incidence, disability-adjusted-life-years averted and costs. Potential future low costs of resistance tests ($30) were used.

**Results:**

The most effective strategy, in terms of DALYs averted, was one using viral load monitoring without confirmation. The incremental cost-effectiveness ratio for this strategy was $2113 (the same as that for viral load monitoring with confirmation). ART monitoring strategies which involved resistance testing did not emerge as being more effective or cost effective than strategies not using it. The slightly reduced ART costs resulting from use of resistance testing, due to less use of second line regimens, was of similar magnitude to the costs of resistance tests.

**Conclusion:**

Use of resistance testing at the time of first line failure as part of the decision whether to switch to second line therapy was not cost-effective, even though the test was assumed to be very inexpensive.

## Introduction

Approximately 10 million people worldwide are currently receiving antiretroviral therapy (ART) [1] which, when effective at suppressing HIV viral replication, is of benefit both in reversing immunodeficiency and reducing infectiousness. WHO guidelines recommend a first line regimen consisting of a non-nucleoside reverse transcriptase inhibitor (NNRTI) efavirenz plus two nucleoside analogue reverse transcriptase inhibitors (NRTI), tenofovir and 3TC/FTC, with a second line regimen consisting of a ritonavir boosted protease inhibitor (bPI) (lopinavir or atazanair) plus two NRTIs (most commonly zidovudine and 3TC/FTC) [2]. Lack of viral suppression in a person who has been on ART over 6 months suggests that either virus with drug resistance has emerged, and possibly was already present at infection, that adherence to the regimen is poor, or a combination of these [3], [4]. If resistance to the NNRTI drug is present there is a clear need to switch to a second line regimen.

There are marked differences between high and low income countries in how people on ART are monitored in order to identify the need to switch treatment. In high income settings, people are monitored with measurement of the plasma HIV viral load at approximately 3–6 monthly intervals and where viral load is not suppressed a drug resistance test is done to detect presence of resistance mutations [5], [6]. If resistance is detected this indicates that a second line regimen is needed. If resistance is not detected then on-going lack of adherence is strongly suspected and interventions to try to improve adherence are recommended [5], [6]. Some guidelines nevertheless recommend switching to a second line regimen in such patients (i.e. a switch is indicated in those with non-suppressed viral load regardless of the result of the resistance test), due to the fact that ritonavir boosted PI regimens are associated with lower risk of resistance than NNRTI regimens in people who are inconsistently adherent [5].

Although guidelines in many countries are changing and viral load testing is being introduced, most low income countries do not yet have access to viral load measures for the majority of people on ART, and resistance testing is hardly available at all. Decisions whether to switch to second line regimens are made based on clinical criteria, if a new WHO stage 3 or 4 condition occurs, or, if available, on the CD4 count [2], both of which are indirect measures of whether the first line regimen remains active. Randomized trials have attempted to ascertain the consequences of providing ART without viral load monitoring [7]–[12]. They have generally found no more than modest differences, both for the outcome of generation of drug resistance and mortality. In contrast, modelling studies have generally indicated that use of viral load monitoring is likely to be associated with some survival benefit, albeit at a level that does not currently make it a cost effective intervention in the most resource limited settings [13]–[20]. This lack of cost-effectiveness of viral load monitoring is due to the cost of current viral load tests but also the cost of second line regimens. Cheaper viral load measurements should become available in the near future, either as point of care tests or using dried blood spots. WHO guidelines recommend that countries adopt viral load monitoring [2], but recognize this should not compromise ART scale-up when treatment gaps exist. Further, costs of second line drugs have been falling, with ritonavir boosted atazanavir now available at $219 per person-year in some settings, representing a drop of around two thirds in cost of a boosted PI within five years [21].

Against this background, it is relevant to consider whether drug resistance testing might have a role in monitoring people on ART in the future in low and middle income settings, if a cheap test could be developed. Such a test might perhaps detect only NNRTI, or NNRTI and NRTI, resistance. Modelling studies conducted in the context of South Africa have suggested that such a test, if used in people for whom a raised viral load is detected to determine whether a switch to second line is required (i.e. switch only made if resistance is detected), would be cost effective, due to the fact that savings in second line drugs would compensate for the cost of the resistance testing [22], [23]. Here we present results from a model of HIV transmission, progression and the effect of ART, which takes account of adherence and resistance in which we compare the effectiveness and cost effectiveness of a range of monitoring strategies which include resistance testing with those that do not. We investigate this under three different contexts of ART monitoring: clinical, with CD4 count or with viral load.

## Methods

### HIV Synthesis Transmission Model

The HIV Synthesis transmission model is an individual-based stochastic model of heterosexual transmission, progression and treatment of HIV infection which incorporates use of specific drugs, resistance mutations, and adherence, and which has been described previously [16], [24], [25]. Further details are provided in the Methods and Results S1, with a detailed model description previously published [25].

### Scenario modelled

We simulated the progression of the HIV epidemic in adults in Zimbabwe up to the beginning of 2015, based on comparisons with data from Demographic and Health Surveys (DHS) and other sources [26]–[29]. We assumed up to 2015 that CD4 counts were used to monitor people on first line, and then considered the introduction of various alternative monitoring strategies after 2015. We compared predicted outcomes over the period 2015 to 2025 in terms of viral suppression, first line failure, switching to second line regimen, death, incidence, disability adjusted life years averted and costs. One single simulation run was used up to 2015. When comparing scenarios from 2015–2025 over 300 runs were made for each strategy and means taken, which effectively eliminates stochastic effects.

**Figure 1:**
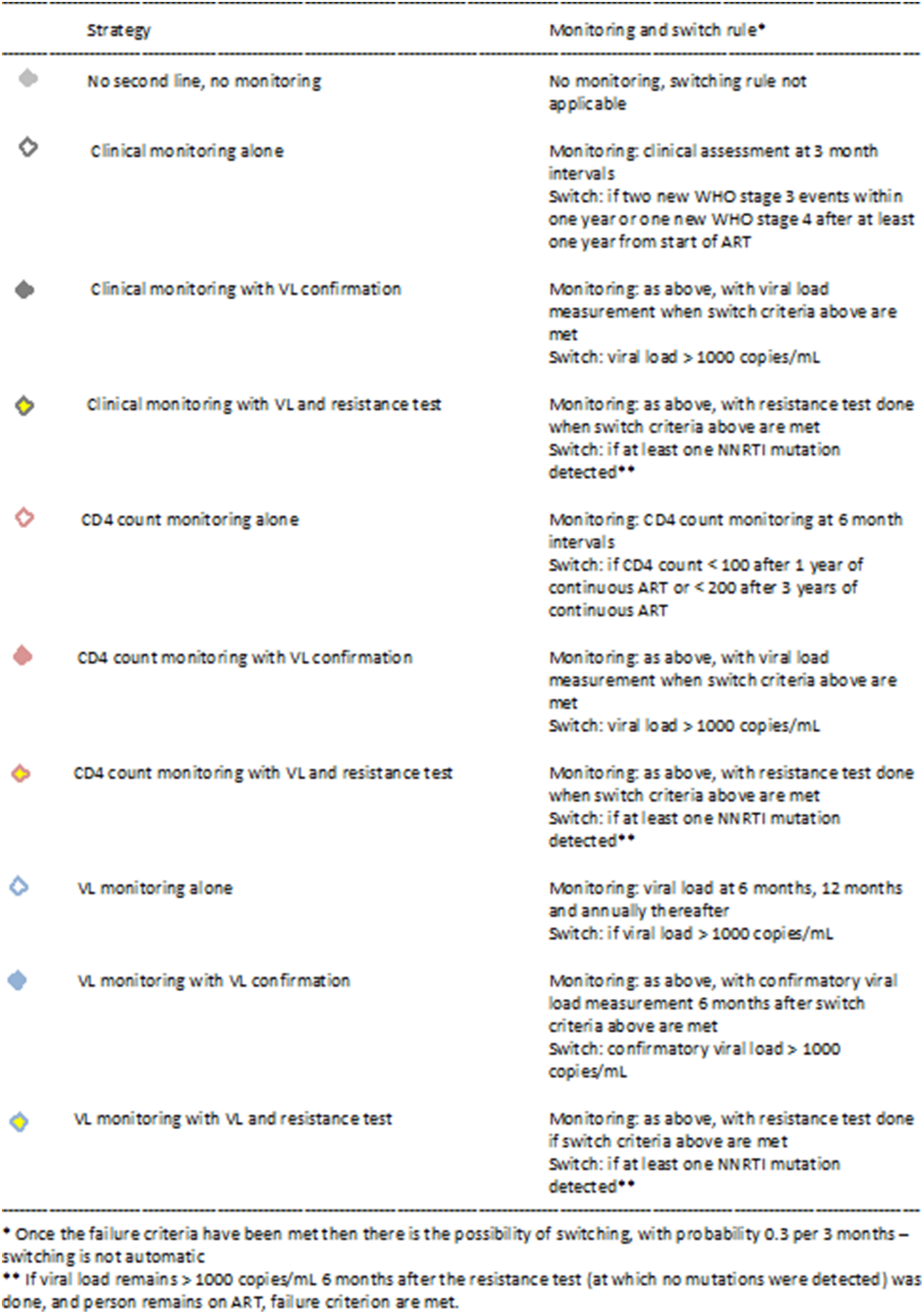
Monitoring strategies and switch criteria.

The evaluated monitoring strategies compared are shown in Figure 1. We classify strategies according to the basic underlying monitoring, which can be clinical (detection of presence of two WHO stage 3 within 1 year or a WHO stage 4 disease, beginning from 1 year after ART initiation), CD4 count-based (6 monthly, beginning 1 year from ART initiation), or viral load-based (6 months, 12 months and then annually). Within each we consider a strategy in which no further measures are performed, one in which a viral load measure is done to confirm failure (with failure declared only if the value is>1000 copies/mL), and one in which both a viral load measure and (if viral load is>1000 copies/mL) a resistance test are done (with failure declared only if the value is>1000 copies/mL and NNRTI resistance is detected). To allow us to get an appropriately broad perspective on alternative monitoring options, we compare them with a reference scenario in which no monitoring is performed and no second line regimen is available. The detection of first line failure does not automatically mean that the person will be switched to second line regimen. We assume that after first line failure, according to whichever strategy is being used, the probability of switching to second line is 0.3 per 3 months. This is much higher than the pre-2015 figure (of 0.03), based on the relatively small numbers of people on second line in Zimbabwe (reflecting that the switch criteria appear to be implemented only very slowly), but was chosen to be similar to the value reported in the UK [30] so that the different effects of the strategies could be fully discerned.

### Economic Analysis

The health benefits associated with the alternative policies were estimated on the basis of disability adjusted life years (DALYs) averted. We assume the objective is to maximize health and that there are no costs incurred with the change in strategy. Costs (presented in 2014 US$) were estimated based upon resource use in the delivery of the policies (see Table S1 in Methods and Results S1). We assumed fully-loaded costs for viral load ($15) and CD4 count ($8) measures in line with what might be hoped to be the costs of available tests in the future, particularly with the availability of point of care alternatives, and of resistance tests ($30). By fully-loaded, we mean inclusion of all costs to the health system required to make the test available and the result delivered to the patient, including personnel and other overhead costs. The time horizon for the analyses is 10 years from 2015 to 2025 (from 2015–2035 in one sensitivity analysis), and both costs and health benefits are discounted to present value using a 3.5% per annum discount rate.

The expected costs and health outcomes (DALYs averted) associated with each of the policy alternatives can be compared to inform which is likely to represent best value from available resources. We present results by plotting the DALYs averted (on the X axis) compared with a policy of no monitoring and no second line, and increment in cost expressed in US dollars (on the Y axis). We draw the cost-effectiveness frontier joining the strategies with most DALYs averted per dollar spent. The slopes of the component lines in the frontier represent the incremental cost effectiveness ratio (ICER) for moving from one strategy to the next most cost-effective option, when moving up and right. To inform the allocation of resources within public health care systems, and thus determine how far along the frontier to go from the origin to choose the optimal strategy one should stop at, it is necessary to know the cost- effectiveness threshold. The cost effectiveness threshold for a country represents the opportunity costs of resources required to fund the intervention, in terms of the health gains those resources could generate if used for alternative purposes in the public health care system [31]. As such, the threshold for a country is not readily apparent, but is likely to be well below $1000 per DALY averted in several countries in sub Saharan Africa, especially when large coverage gaps for ART and other basic interventions exist. Health utilities/disability weights to calculate DALYs averted were derived from a recent comprehensive study [32]. Several one way sensitivity analyses were performed to examine the influence of various parameter values.

## Results

### Status of the population in 2015

Table 1 shows the situation for the simulated population at the beginning of 2015, the year from which the various alternative monitoring strategies are compared. Of those on ART, a high proportion (85%) have viral load <500 cps/mL, 13% have failed first line, according to the CD4 count monitoring strategy assumed to be used before 2015, but only 7% have started second line, due to a low rate of switch in those with first line failure. Of those on ART with viral load >500, 76% have NNRTI resistance.

**Table 1:** Characteristics of the population at baseline at beginning of 2015 (adults 15–65 years old).

Indicator		Data sources*
HIV incidence (per 100 personyears)	0.61	0.67 in 2011 Spectrum [27]
HIV prevalence (age 15–45)	11%	15% in 2011 DHS [28]
% with transmitted NNRTIresistance at ART initiation	9%	3%–22% (2008–2010) [39]
% diagnosed	89%	Inferred based on 550,000 adults on ART in 2012(∼50% of all HIV+) [27], [29]
Of diagnosed, % on ART	66%	As above
Of diagnosed, % ART experienced	76%	Percentage of adults and children with HIV knownto be on treatment 12 months after initiating antiretroviraltherapy 85.7% according to the NAC October 2009 Cohortdata that was analysed in 2010 [27]
% of all HIV+ on ART	59%	550,000 adults on ART in 2012 (∼50% of all HIV+)[27], [29]
% of people on ART with VL <500	85%	[39]
% of ART experienced peoplewith VL <500	77%	[39]
% of those on ART failed first line	13%	No data found
% of ART experienced peoplewho started second line	7%	WHO reports 4% in LMICs
Of those on ART with viralload>500, % with NNRTIresistance	76%	[39]

*note the data are given to enable comparison with simulated indicators – model is not formally calibrated to observed data in the references.

### Predicted outcomes according to monitoring strategy

**Figure 2:**
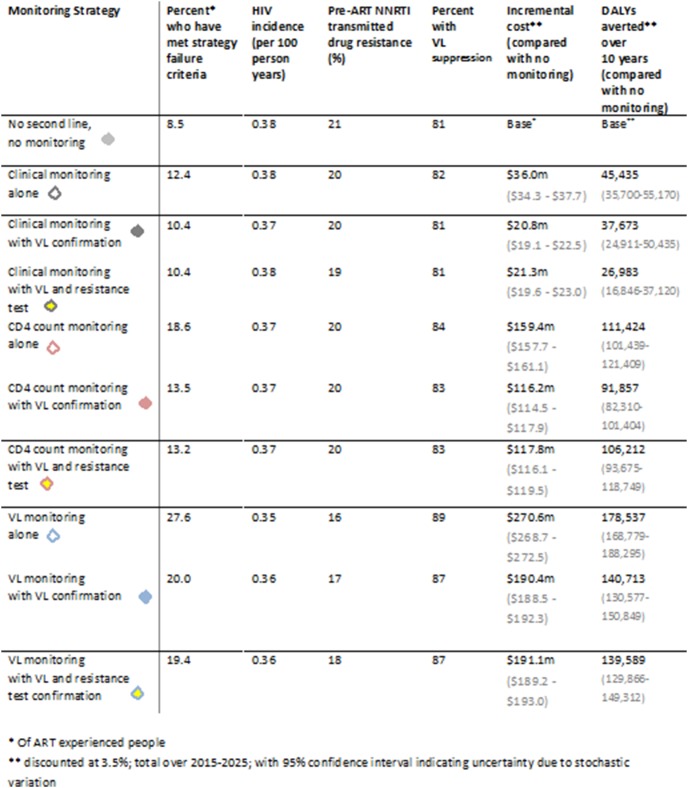
Outcomes by monitoring strategy. Mean over 2015–2025 (except for cost and DALYs where total over this period is given). VL - viral load.

Figure 2 shows outcomes (mean over 2015–2025) according to monitoring strategy. The proportion of ART-experienced people identified as having failed first line, according to the specific criteria for the strategy, is generally highest with the viral load monitoring strategies, intermediate with the CD4 count monitoring strategies, and lowest with the clinical monitoring strategies. The proportion who failed first line in the strategy where there is no monitoring from 2015 is a reflection of those who had already failed by 2015 and remain alive. Use of viral load to confirm failure results in a lower proportion identified as failing first line, but there is relatively little difference with additional confirmation with a resistance test.

The proportion of people with viral suppression is predicted to be highest with viral load monitoring without confirmation. The mean proportion of people initiating ART who have transmitted drug resistance is predicted to be lowest with use of viral load monitoring, but the additional use of the resistance test does not result in a lower proportion. HIV incidence in the population is not predicted to differ much by strategy although it is lowest with the viral load monitoring strategy (without confirmation with a second viral load). The total discounted DALYs averted is also shown, highest for the viral load monitoring strategy without confirmation.

### Costs of strategies

**Figure 3:**
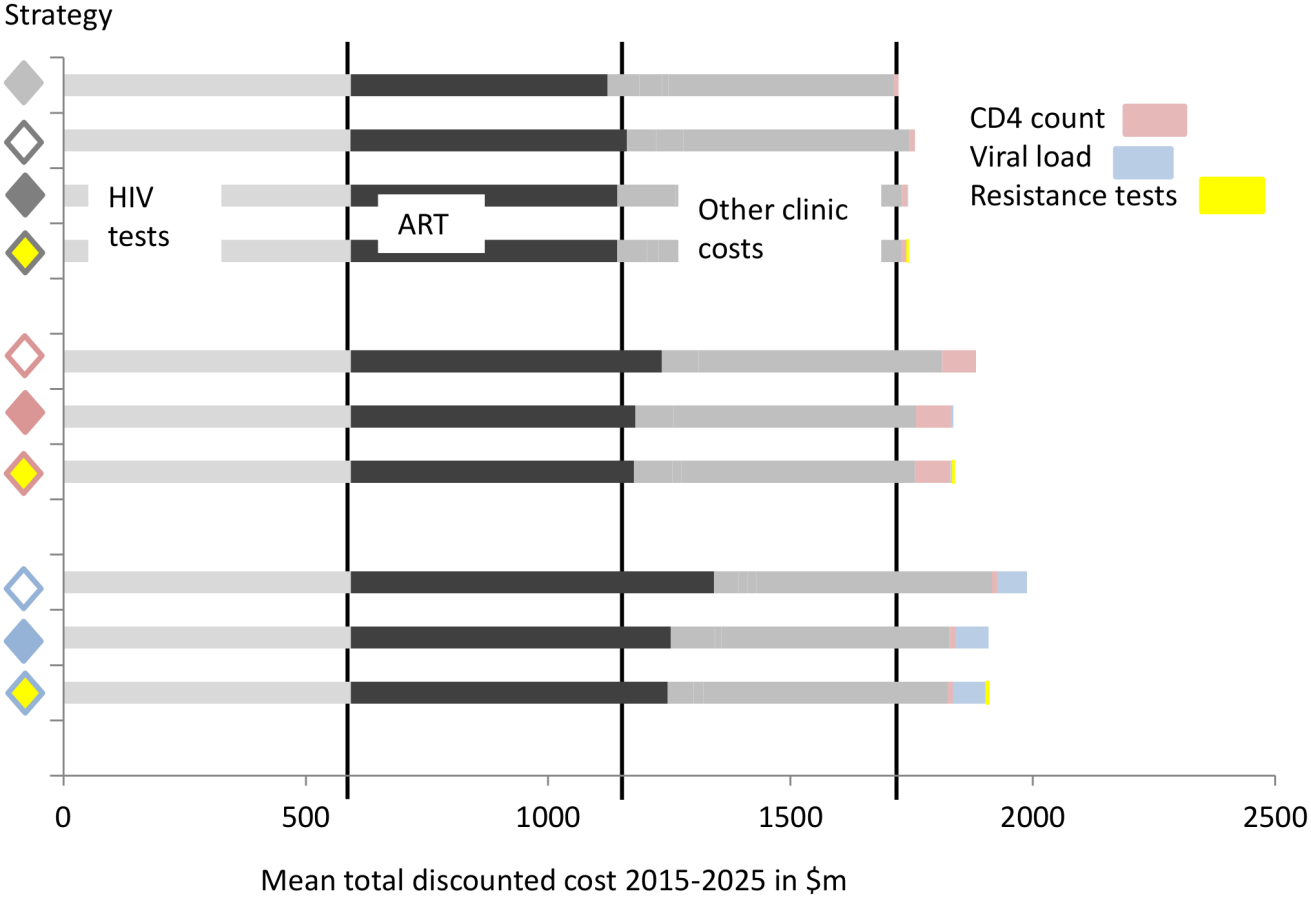
Breakdown of costs by strategy - total discounted cost 2015–2025, in $million. See Figure 1 for legend for strategies.

Figure 3 shows the breakdown of costs according to strategy, which indicates that, at the low unit costs assumed, monitoring costs make up a relatively small proportion of all HIV programme costs, with viral load test costs being 4% of total treatment and care costs for viral load monitoring-based strategies, and resistance test costs, for strategies involving their use, being below 1%. Focussing on the comparison between the strategy with viral load monitoring (with viral load confirmation) with that using viral load monitoring with both viral load and resistance testing, the reduction in ART cost with the use of resistance testing is around $6 m, while the cost of the resistance tests themselves is $4.5 m, making the overall costs very similar. The increment in total cost for each strategy compared with the reference strategy is shown in Figure 2.

### Cost effective frontier

**Figure 4:**
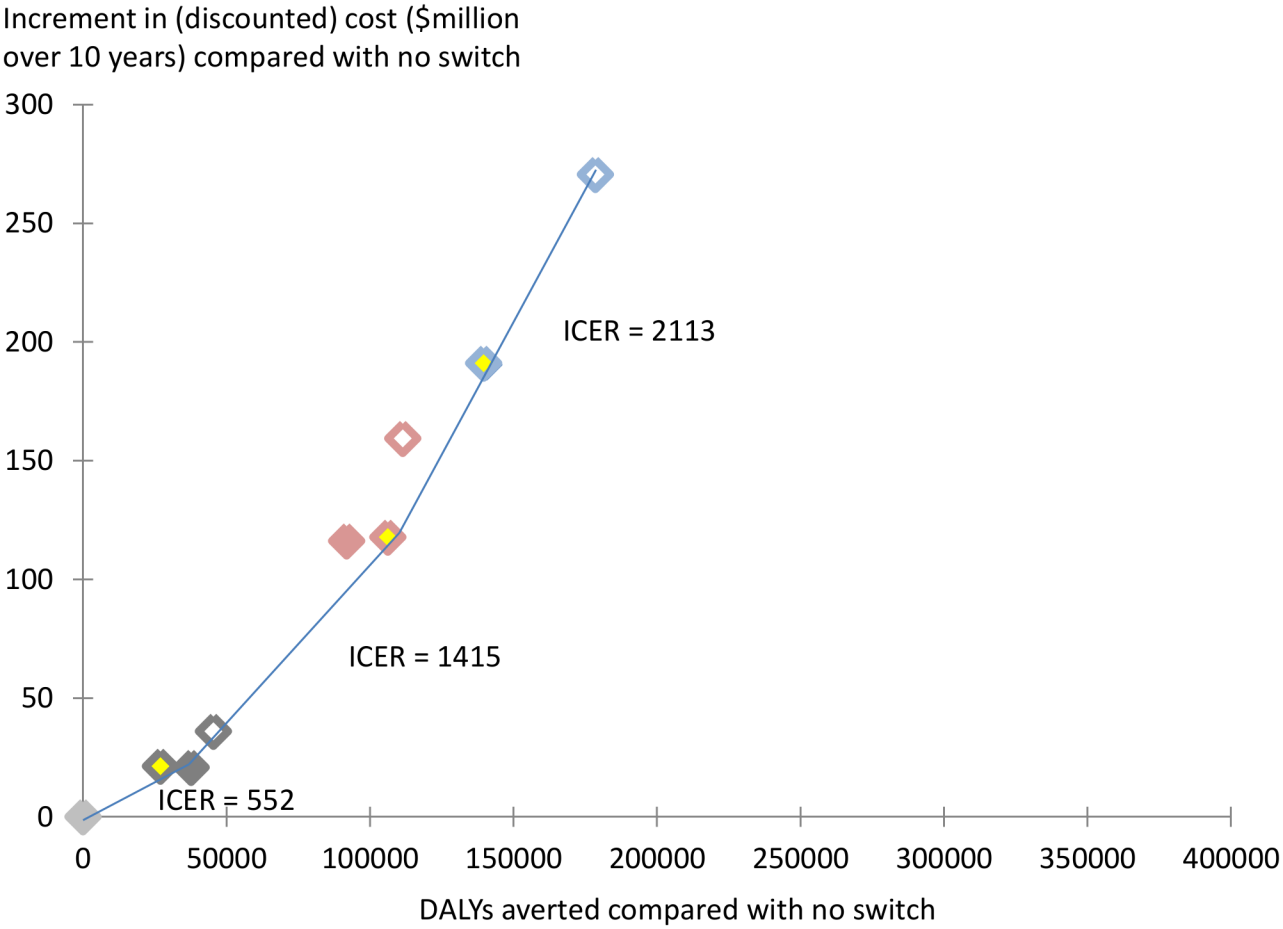
Incremental costs and DALYs averted for monitoring strategies over 10 years, compared with no monitoring, no second line. See Figure 1 for legend for strategies.

Figure 4 shows the incremental costs and DALYs averted compared with the strategy of no monitoring and no second line availability. Generally, strategies that are based on viral load monitoring avert most DALYs, followed by strategies based on CD4 count monitoring while strategies based on clinical monitoring result in least DALYs averted. However, strategies involving use of resistance testing to confirm failure do not appear to offer clear advantages compared with strategies that do not. In this assessment, viral load monitoring, with confirmation, becomes cost effective at a cost effectiveness threshold of $2113, with further DALYs averted at the same ICER with use of a single viral load without confirmation.

In a further analysis we concentrated on the comparison between the strategy with viral load monitoring (with viral load confirmation) with that using viral load monitoring with both viral load and resistance testing. For those with confirmed viral load >1000 cps/mL but no resistance present we compared the status after 1 year, according to these two strategies. Without resistance testing the proportions who (i) remained on first line ART and had viral load <1000 copies/mL, (ii) remained on first line ART and had viral load ≥1000 copies/mL, (iii) had started second line ART and had with viral load <1000 copies/mL, (iv) remained on first line ART and had viral load ≥1000 copies/mL, (vi) were off ART, or (vi) were dead, were, respectively, 5%, 20%, 46%, 8%, 10% and 10% (i.e. 51% on ART with viral load <1000 copies/mL). The corresponding percentages using resistance test confirmation were 39%, 23%, 12%, 6%, 11% and 10% (i.e. the proportion on ART with viral load <1000 copies/mL is similar).

### Sensitivity analyses

**Figure 5:**
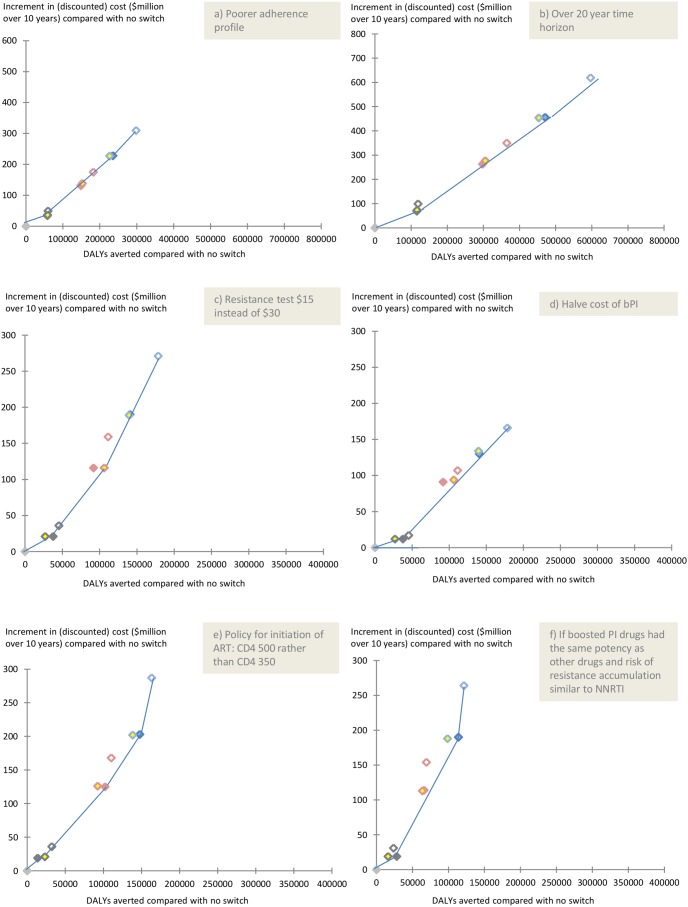
One way sensitivity analyses with the following changes from the base scenario (a) poorer adherence profile (b) 20 year time horizon (c) resistance test cost of $15 instead of $30 (d) cost of second line drugs made the same as cost of 1^st^ line (e) initiation of ART at CD4 count below 500 cells/mm^3^ rather than below 350 cells/mm^3^ (f) if boosted PI drugs had same potency as other drugs, and risk of resistance accumulation similar to NNRTI.

We conducted several one way sensitivity analyses to see if the main findings would differ, when varying the assumptions most likely to influence these results (Figure 5). In none of the following situations did the overall conclusions change: (a) a poorer overall population adherence profile (so that only 76% had on average an adherence above 80%, compared with 89% in the main analysis) (b) a 20 year time horizon (c) a resistance test cost of $15 instead of $30 (d) with the cost of bPI halved and (e) initiation of ART at CD4 count below 500/mm^3^ rather than 350/mm^3^ and considering a scenario where boosted PI drugs have the same (instead of higher, as in base case) potency as other drugs, and risk of resistance accumulation is similar to NNRTI drugs (rather than lower, as in base case). Results from further sensitivity analyses are shown in Figure S1 of Methods and Results S1.

## Discussion

We evaluated whether availability of a relatively cheap drug resistance test might appreciably increase the effectiveness of adult ART monitoring in low income settings and be cost effective. We were unable to identify that this would be the case. This is in contrast to the conclusion reached in modelling studies conducted by Rosen et al [22] and Levison et al [23], who concluded that use of resistance testing at first line virologic failure would be cost effective in South Africa and potentially also in lower income settings, due to the fact that people with no drug resistance would not be put onto expensive second line boosted PI regimens. The difference in findings partially relates to differences in modelled outcomes for people who do not have resistance detected at first line failure. Levison et al assume that a proportion of patients in this situation subsequently achieve durable viral suppression on first line ART and thereafter have the same outcomes as those with initial virologic success [23]. Our model predicts that for a person with virologic failure but no resistance mutations outcomes are generally better if a switch to second line is made than if it is not. This prediction is due to the strong person-specific component to adherence, the fact that the improvement in adherence that is triggered by a high viral load measurement may be temporary, and the fact that the second line regimen is more forgiving of intermittent adherence because the rate of emergence of resistance to ritonavir boosted protease inhibitors is low [33], [34] ^33,34^ and potency is high, given the ability to suppress viral load when used as a single drug (albeit not to the same extent as a triple combination of drugs) [35]. The prediction is consistent with the recommended course of action in some treatment guidelines [5] but there is little direct evidence. Data on outcomes for people who have a non-suppressed viral load but no resistance mutations detected, according to whether a switch is made to second line, would be useful for further informing models in this area. Ideally, a randomized trial might be performed.

The pattern of our results was generally similar in sensitivity analyses (Figure 5). When considering a 20 year time horizon (instead of 10) and of a situation in which adherence levels were lower, differences in DALYs averted between strategies increased, but the relative ordering generally remained very similar.

We assumed that if a resistance test is performed in a person failing a first line regimen then the decision whether to switch would be based on whether NNRTI resistance is present. One could consider also basing the decision on presence of specific nucleoside reverse transcriptase inhibitor mutations, for example to tenofovir or zidovudine, but this would seem unlikely to change our results since NNRTI mutations tend to appear most readily. While our model does not suggest a key role for resistance tests in monitoring of people on ART to decide on switching from first to second line regimens, there are other future potential applications of resistance tests which should be considered and which mean that development of cheap, perhaps point of care, resistance test might be valuable. For example, as the proportion of attendees at ART initiation clinics who have NNRTI drug resistance grows, either due to transmitted drug resistance or possibly previous undeclared ART use, it could become cost effective to perform individual level resistance testing before therapy initiation to decide on which starting regimen to use in individuals, perhaps at least in some middle income settings. Another modelling study has previously suggested that such testing would not be cost effective unless levels of transmitted drug resistance are very high [36]. At least one trial is on-going in this area, in Kenya [37]. There could also be a future role of resistance testing in patients failing second line regimens. Further, there could well be a more cost-effective role for resistance testing in routine care in selecting drug regimens for pregnant women who are not on ART at pregnancy presentation, since it is important to select a regimen that will maximise the chance of viral suppression at the time of birth. Again, this was not formally evaluated here.

We assumed that monitoring strategies would be carried out perfectly, so that all people in care are monitored as indicated in the guidelines, that all measurement results are returned to the clinic within the necessary time, that the health workers responsible for monitoring people on ART correctly implement the switch algorithm, and that the probability of switch given first line failure is independent of the failure definition. In future work comparing monitoring strategies it will be important to assess the impact of real life challenges with implementation as this may result in favouring of simpler more robust monitoring approaches. So far, rates of switching to second line regimens have been low in African settings [38]. Further, although our model is extensively calibrated to multiple data sources to ensure that we capture the dual influences of adherence and resistance on viral load level, as with any modelling analysis there is the possibility that this does not fully capture the critical elements of the underlying process necessary to reach correct conclusions over the comparisons made. As further data emerges we can revisit this question, if necessary amending our model as newly emerging data point to any mis-specified elements.

In conclusion, we did not identify a compelling role for drug resistance testing as part of ART monitoring in making the decision whether to switch from first to second line.

## Supporting Information

Methods and Results S1 **Supplementary methods, tables and figures, and parameter values and costs.**
(DOC)
