# Correction: Cost-Effectiveness of HIV Drug Resistance Testing to Inform Switching to Second Line Antiretroviral Therapy in Low Income Settings

**DOI:** 10.1371/journal.pone.0115079

**Published:** 2014-12-01

**Authors:** The PLOS ONE Staff

The fourth author’s name is spelled incorrectly. The correct name is: Travor Mabugu. The correct citation is: Phillips A, Cambiano V, Nakagawa F, Mabugu T, Miners A, et al. (2014) Cost-Effectiveness of HIV Drug Resistance Testing to Inform Switching to Second Line Antiretroviral Therapy in Low Income Settings. PLoS ONE 9(10): e109148. doi:10.1371/journal.pone.0109148.
