# Supplementary material for: Cost-Effectiveness of HIV Drug Resistance Testing to Inform Switching to Second Line Antiretroviral Therapy in Low Income Settings
Source: PLoS One. 2014 Oct 7;9(10):e109148. doi: 10.1371/journal.pone.0109148 (PMC4188574; doi:10.1371/journal.pone.0109148)
Supplement: Methods and Results S1 — Supplementary methods, tables and figures, and parameter values and costs. (DOC) [file pone.0109148.s001.doc]

Cost-effectiveness of HIV drug resistance testing to inform switching to second line antiretroviral therapy in low income settings

Supplementary Methods and Results

Contents

1. Supplementary Methods Page 2
2. References - Supplementary Page 6
3. Supplementary tables and figures Page 8
4. Parameter values and costs Page 9

Supplementary Methods

This section provides further detail of the modelling performed. In order for this to be a complete description in itself there is some repetition of the main paper in places.

*HIV Synthesis Transmission Model*

The HIV Synthesis transmission model is an individual-based stochastic model of heterosexual transmission, progression and treatment of HIV infection within a southern African context^1-3^. Full updated model details are in previously published supplementary material^2^, but a brief description follows. Resistance is modelled in terms of the presence or absence of specific mutations, with consideration given to the effect of such mutations on virus susceptibility to specific drugs. Distinction is made for each mutation as to whether it is only present in minority virus, so assumed not transmissible, or if it is present in majority virus, and hence assumed transmissible. The model has been employed to contribute to several HIV Modelling Consortium joint modelling projects^4^.

All variables are updated in 3 month periods in the model, which includes an age- and gender- structure. Sexual risk behaviour is modeled as the number of condomless-sex short term partners and presence of a condomless-sex long-term partner in each period. Emergence of resistance on ART is dependent on adherence and the current number of active drugs. Adherence is assumed to have two main components, a lifelong tendency to adhere for each individual, and a period to period variation. In addition, various factors can influence adherence, including the initial measurement of viral load > 1000 copies/mL which is assumed to lead to an increase in adherence in 70% of people as a result of targeted adherence intervention; this is consistent with data showing that a high proportion of people with measured viral load > 1000 copies/mL who undergo an adherence intervention subsequently achieve viral suppression without a change in ART ^5,6^ and broadly consistent with a meta-analysis^7^. Although the appropriate duration to assume for this effect is uncertain^8^, the impact of adherence interventions has often been shown to diminish with time^9^. Based on this overall body of data, we assume that the adherence intervention is effective only the first time it is performed and that for 40% the effect is permanent (28% of those with a viral load >1000), but that in the remaining 60% (42% of those with viral load>1000) it lasts only 6 months. We also model the possibility that in some individuals at certain times the adherence is so low that the individual has in fact interrupted ART, although this is undeclared to the clinic so the person is still considered by the clinic in any records kept as being on ART. Such a lack of adherence is thought to explain why some people have no resistance mutations present at virologic failure^10,11^, and we capture this phenomenon. Regarding resistance and transmission, the presence or not of resistance mutations does not influence the risk of transmission (i.e. virus with resistance mutations present is assumed equally transmissible as virus without such mutations, for a given viral load). Resistance is modelled in terms of the presence or absence of mutations specific to the drugs in use. Distinction is made for each mutation as to whether it is only present in minority virus (if the patient has a mutation present but has stopped drugs that select for that mutation), so the mutation is assumed not transmissible, or if it is present in majority virus, and hence the mutation is assumed transmissible. The probability that resistance mutations present in majority virus of the source partner are transmitted to the newly infected person is dependent on the specific mutation. Once a resistance mutation is transmitted to the new host it is assumed to have a certain probability of being lost from majority virus over time ^12^. Even after being lost from majority virus, it is assumed to remain in minority virus and is selected back as majority virus if an ART selecting for that mutation is initiated. We also consider the possibility of a person who is already infected become super-infected, including with drug resistant HIV^13^, although there is assumed to be at most a 20% chance that a person super-infected by a person with HIV resistance then has virus with those resistance mutations as a result.

*Scenario modelled*

We simulated the progression of the HIV epidemic in Zimbabwe up to the beginning of 2015, based on comparison with data from Demographic and Health Surveys (DHS) and other sources^14-17^.  We model adult sexual behaviour and HIV infections occuring in adults up to any age, although risk of acquisition of HIV is not considered after age 65. We assume up to 2015 that the CD4 count was used to monitor people on first line, and then considered the introduction of various alternative monitoring strategies after 2015. We compared predicted outcomes after 2015 in terms of viral suppression, first line failure, switching to second line regimen, death, incidence, and disability adjusted life years averted and costs. One single simulation run was used up to 2015 as, given the calibration to data from Zimbabwe, we felt it unlikely that results would be affected by the exact choice of parameter values, or exact realization of an epidemic for a given set of parameter values . When comparing scenarios from 2015-2025 over 300 runs were made for each strategy and means taken, which effectively eliminates stochastic effects.

The evaluated monitoring strategies compared are shown in table 1 of the main paper. We classify strategies according to the basic underlying monitoring, which can be clinical (detection of presence of two WHO stage 3 within 1 year or a WHO stage 4 disease, beginning from 1 year after ART initiation), CD4 count-based (6 monthly, beginning 1 year from ART initiation), or viral load-based (6 months, 12 months and then annually). Within each we consider a strategy in which no further measures are performed, one in which a viral load measure is done to confirm failure (with failure declared only if the value is > 1000 copies/mL), and one in which both a viral load measure and (if viral load is above 1000 copies/mL) a resistance test are done (with failure declared only if the value is > 1000 copies/mL and NNRTI resistance is detected). We compare them with a reference scenario in which no monitoring is performed and no second line regimen is available.

We assume that after first line failure, according to whichever strategy is being used, the probability of switching to second line is 0.3 per 3 months. This is much higher than the pre-2015 figure (of 0.03), based on the relatively small numbers of people on second line (reflecting that the switch criteria appear to be implemented only very slowly), but was chosen so that the different effects of the strategies could be fully discerned.

*Economic Analysis*

The health benefits associated with the alternative policies were estimated on the basis of disability adjusted life years (DALYs) averted. We assume the objective is to maximize health and that there are no costs incurred with the change in strategy. Costs (presented in 2014 US$) were estimated based upon resource use in the delivery of the policies (see below). We assumed fully-loaded costs for viral load ($15) and CD4 count ($8) measures in line with what might be hoped to be the costs of available tests in the future and of resistance tests ($30) in line with what might be the minimum attainable fully-loaded cost of providing a new assay designed for resource limited settings. By fully-loaded, we mean inclusion of all costs to the health system required to make the test available and the result delivered to the patient, including personnel and other overhead costs. Introduction of laboratory-based viral load monitoring and resistance testing would require significant capital investment that may not be necessary if point of care alternatives become available in the near future. The time horizon for the analyses is 10 years from 2015 to 2025 (from 2015-2035 in one sensitivity analysis), and both costs and health benefits are discounted to present value using a 3.5% per annum discount rate.

The expected costs and health outcomes (DALYs averted) associated with each of the policy alternatives can be compared to inform which is likely to represent best value from available resources. We present results by plotting the DALYs averted (on the X axis), compared with a policy of no monitoring, no second line, and increment in cost in $ on the Y axis. We draw the efficiency frontier; the most productively efficient strategies – most DALYs averted per $ spent - lie on this line. The slopes of the component lines on the frontier represent the incremental cost effectiveness ratio (ICER) for moving from one strategy to the next most cost-effective option, when moving up and right. To inform the allocation of resources within public health care systems, and thus determine how far along the line to go from the origin to choose the optimal strategy one should stop at, requires a cost- effectiveness threshold. The cost effectiveness threshold for a country represents the opportunity costs of resources required to fund the intervention, in terms of the health gains those resources could generate if used for alternative purposes in the public health care system^18^. As such, the threshold for a country is not readily apparent, but is likely to be well below $1000 per DALY averted in several countries in sub Saharan Africa, especially when large coverage gaps for ART and other basic interventions exist. Health utilities / disability weights were derived from a recent comprehensive study^19^.

Several one way sensitivity analyses were performed to examine the influence of various parameter values. In the main paper results we considered (a) a poorer overall population adherence profile (b) a 20 year time horizon (c) a resistance test cost of $15 instead of $30 (d) with the cost of bPI halved and (e) initiation of ART at CD4 count below 500 /mm^3^ rather than 350 /mm^3^. In this Supplementary results section we further considered scenarios such that (a) people starting second line on average have a decrease in adherence, due to gastrointestinal adverse effects of boosted PI (b) people starting second line on average have an increase in adherence, due to greater motivation to adhere (c) decreased probabiity of switch once failure criteria fulfilled (d) immediate switch once failure criteria fulfilled (e) decreased death rate in people with current WHO stage 4 disease or TB (f) decreased underlying variability in CD4 count (g) Increased underlying variability in CD4 count (h) decreased extent to which people interrupt ART with the clinic unaware (and so are considered on ART but have no resistance mutations) (i) increased extent to which people interrupt ART with the clinic unaware (j) decreased rate of treatment interruption (k) increased rate of treatment interruption (l) situation in which boosted PI drugs had half potency of other drugs (so preserving activity of nucleosides more important).

**References - Supplementary Methods and Results**

1. Phillips AN, Pillay D, Garnett G, et al. Effect on transmission of HIV-1 resistance of timing of implementation of viral load monitoring to determine switches from first to second-line regimens in resource-limited settings. AIDS 2011; 25: 843–50.
2. Cambiano V, Bertagnolio S, Jordan M, et al. Transmission of Drug Resistant HIV and Its Potential Impact on Mortality and Treatment Outcomes in Resource-Limited Settings. J Infect Dis 2013; 207: S57-62
3. Cambiano V, Bertagnolio S, Jordan M, Pillay D, Perriens J, Venter F, et al. Predicted levels of HIV drug resistance: potential impact of expanding diagnosis, retention, and eligibility criteria for antiretroviral therapy initiation. AIDS 2014, 28 (Suppl 1)*:*S15–S23.
4. Keebler D, Revill P, Braithewaite S, et al. Use of HIV programmes to monitor adults on antiretroviral treatment: a combined analysis of three mathematical models. Lancet Global Health 2013.
5. Orrell C, Harling G, Lawn SD et al. Conservation offirst-line antiretroviral treatment regimen where therapeuticoptions are limited. Antiviral Therapy 2007; 12:83–88.
6. Hoffmann CJ, Charalambous S, Sim J, et al. Viremia, Resuppression, and Time to Resistance in Human Immunodeficiency Virus (HIV) Subtype C during First-Line Antiretroviral Therapy in South Africa. Clin Infect Dis 2013; 2009; 49:1928–35.
7. Bonner K, Mezochow A, Roberts T, et al. Viral Load Monitoring as a Tool to Reinforce Adherence: A Systematic Review. J Acquir Immune Defic Syndr 2013;64:74–78.
8. Hoffmann CJ, Charalambous S, Grant AD, Morris L, Churchyard GJ, Chaisson RE. Durable HIV RNA resuppression after virologic failure while remaining on a first-line regimen: a cohort study. Trop Med & Int Health 2013
9. Bärnighausen T, Chaiyachati K, Chimbindi N, et al. Interventions to increase antiretroviral adherence in sub-Saharan Africa: a systematic review of evaluation studies. Lancet Infect Dis 2011; 11: 942–51.
10. Gupta R, Hill A,Sawyer AW, Pillay D. Emergence of Drug Resistance in HIV Type 1–Infected Patients after Receipt of First-Line Highly Active Antiretroviral Therapy: A Systematic Review of Clinical Trials. Clinical Infectious Diseases 2008; 47:712–722
11. Johnston V, Cohen K, Weisner L, Morris L, Ledwaba J, Fielding K, Charalambous S, Churchyard G, Phillips AN, Grant A. Viral suppression following switch to second-line antiretroviral therapy: the role of resistance and ‘sub-therapeutic’ drug concentrations prior to switch. JAIDS 2013.
12. Castro H, Pillay D, Cane P, Asboe A, Cambiano V, Phillips AN, Dunn DT. Persistence of Transmitted HIV-1 Drug Resistance Mutations. JID 2013; DOI: 10.1093/infdis/jit345
13. Smith DM, Wong JK, Hightower GK, et al. HIV drug resistance acquired through superinfection. AIDS 2005; **19:** 1251–56.
14. Gregson S, Gonese E, Hallett TB, et al. HIV decline in Zimbabwe due to reductions in risky sex? Evidence from a comprehensive epidemiological review. Int J Epid 2010;39:1311–1323.
15. UNAIDS. Global AIDS response progress report 2012. Follow up to the 2011 Political Declaration on HIV/AIDS: Intensifying our efforts to eliminate HIV/AIDS. Zimbabwe Country Report. Reporting Period: January 2010-December 2011. <http://www.unaids.org/en/dataanalysis/knowyourresponse/countryprogressreports/2012countries/ce_ZW_Narrative_Report.pdf>
16. Zimbabwe National Statistics Agency (ZIMSTAT) and ICF International. 2012. Zimbabwe Demographic and Health Survey 2010-11. Calverton, Maryland: ZIMSTAT and ICF International Inc.
17. Global Fund to Fight HIV, TB and Malaria. Zimbabwe Quarterly reports.
18. Claxton, K., Walker, S., Palmer, S., Sculpher, M., ‘Appropriate Perspectives for Health Care Decisions’, Centre for Health Economics Research Paper 54, University of York, 2010.
19. Salomon JA, Vos T, Hogan DR, et al. Common values in assessing health outcomes from disease and injury: disability weights measurement study for the Global Burden of Disease Study 2010. Lancet 2012; 380: 2129–4310.

Supplementary Tables and Figures

**Supplementary Table S1**. Costs and utilities assumed. (costs of drugs per person year)

**Supplementary Figure S1.** One way sensitivity analyses with the following changes from the base scenario (a) People starting second line on average have a decrease in adherence, due to gastrointestinal adverse effects of boosted PI (b) People starting second line on average have an increase in adherence, due to greater motivation to adhere (c) Decreased probability of switch once failure criteria fulfilled (d) Immediate switch once failure criteria fulfilled (e) Decreased death rate in people with current WHO stage 4 disease or TB (f) Decreased underlying variability in CD4 count (g) Increased underlying variability in CD4 count (h) Decreased extent to which people interrupt ART with the clinic unaware (and so are considered on ART but have no resistance mutations) (i) Increased extent to which people interrupt ART with the clinic unaware (j) Decreased rate of treatment interruption (k) Increased rate of treatment interruption (l) Situation in which boosted PI drugs had half potency of other drugs (so preserving activity of nucleosides more important).

Parameter values and costs

**Parameter values for main analysis and distributions used for uncertainty analysis**

Details of variables are explained in Model details in Supplement to ref 2.

-----------------------------------------------------------------------------------------------------------------------------------------------

**Sexual behaviour**

Sexual behaviour model structure: base structure (*rbm*=4; see model details)

Change in propensity to have a long term condomless sex partner after HIV diagnosis (ch_risk_diag): 9/13

Change in propensity to have short term (“new”) condomless sex partners after HIV diagnosis (ch_risk_diag_newp): 5/6

Date at which population level change in condomless sex behaviour occurs (date_ch_risk_beh): 1995

Change in propensity to have condomless sex (“risk behaviour”) with short term partners after threshold for population level change in condomless sex behaviour reached (ch_risk_beh_newp): 0.2

Change in propensity to have a long term condomless sex (“risk behaviour”) with short term partners after threshold for population level change in condomless sex behaviour reached (ch_risk_beh_ep): 0.5

Rate of starting new long term condomless sex partnership in 15-25 year age group (eprate): 0.1

Poisson mean for moderately high short term partner group (see model details) (highsa): 4.3

Poisson mean for highest short term partner group (see model details) (swn):7

Factor to change overall average level of condomless sex with short term partners (newp_factor): 9

Proportion of the population who have a lifetime reduced number of condomless sex partners (see model details) (p_rred_p): 0.20

Probability per 3 months of pregnency at age 35-45 (prob_pregnancy_base): 0.037

Fold difference in pregnancy rate at age: 1525: 1.04; 25-35: 1.03; 45-55: 0.975

-----------------------------------------------------------------------------------------------------------------------------------------------

**Transmission**

Fold difference in transmission rate for a given viral load (see Model details for base assumption on transmission rate by viral load^2^): 1.0

Rate of transmission in primary HIV infection (lasting 3 months) (tr_rate_primary): 0.25

Transmission rate when plasma viral load is < 500 cps/mL (tr_rate_undetec_vl): 0.001

Fold higher rate of transmission from men to women, compared with women to men (fold_change_w): 1.5

Fold higher rate of transmission in young women compared with older women (fold_change_yw): 2.0

Fold higher rate of transmission if current STI present (fold_change_sti): 3.0

Fold lower transmission rate for short term partners compared with long term (reflecting average lower number of sex acts) (fold_tr_newp): 5/14

Super-infection: for people with HIV super-infected with a resistant virus, we assume a low (20%) probability that these mutations are established as resistance mutations.

Adjustment to factor determining extent to which some transmitted resistance is effectively immediately lost (even from minority virus) (res_trans_factor): 0.75

Probability per 3 months of loss of persistence of transmitetd mutations from majority virus to minority virus (same for each mutation) (rate_loss_persistence): 0.04

Probability per 3 months of loss of NNRTI mutations, acquired due to PMTCT, from majority virus to become only in minority virus (rate_loss_nnres_pmtct_maj): 0.25

Probability per 3 months of loss of virus with NNRTI mutations acquired due to PMTCT, from minority virus to effectively be extinct altogether (rate_loss_nnres_pmtct_min): 0.25

Prevalence of male circumcision: 0.1

Date of introduction of voluntary male medical circumcision (VMMC): 2008

Rate of increase in probability of VMMC per 3 months: (circ_inc_rate): 0.003

-----------------------------------------------------------------------------------------------------------------------------------------------

**HIV testing**

Date start of testing (date_start_testing): 1996

Initial test probability for those with WHO condition (this increases by 0.008 per 3 mths after testing is introduced, up to 2015) (test_rate_who4): 0.2

Initial test probability for those with TB (this increases by 0.005 per 3 mths after testing is introduced, up to 2015) (test_rate_tb): 0.1

Initial test probability for those with current WHO 3 condition (this increases by 0.0012 per 3 mths after testing is introduced, up to 2015) (test_rate_who3): 0.03

Reduction in rate of testing if never had condomless sex (red_test_neversex): 0.33

Annual linear increase in testing (an_lin_incr_test): 0.001

Date start testing ANC (date_start_testanc): 1994

Rate test ANC (rate_testanc_inc): =0.0025

Fold difference in ANC testing rate by age:

fold_probanc_1519=0.73, fold_probanc_2024=1x1.36,,fold_probanc_2529=0.9x1.14, fold_probanc_3039=0.8x1.00, fold_probanc_4049=0.70, fold_probanc_ov50=0.56

-----------------------------------------------------------------------------------------------------------------------------------------------

**Natural progression**

Probability of being lost (unlinked to care) at diagnosis (prob_loss_at_diag): 0.6

Initial CD4 count at infection (square root scale) (mean_sqrtcd4_inf): 27.5

Factor adjusting basic rate of natural cd4 decline (see model details) (fx): 1.0

Factor adjusting basic rate of natural viral load change (see model details) (gx): 1.0

Fold increase in risk of WHO 3 condition, compared with risk of WHO 4 condition, for given level of CD4 count, viral load and age (fold_incr_who3): 5

Fold decrease in risk of HIV-related death, compared with risk of WHO 4 condition, for given level of CD4 count, viral load and age (fold_decr_hivdeath): 0.25

Fold difference in risk of WHO 4 condition, for given level of CD4 count, viral load and age, compared with base assumption (see model details) (fold_change_in_risk_base_rate): 1.0

Increase in death rate in 3 months period in which a WHO 4 condition is present (incr_death_rate_adc): 5

Increase in death rate in 3 months period in which TB is present incr_death_rate_tb): 2

Fold difference in non HIV related mortality, compared with base assumption (fold_change_ac_death_rate): 1

-----------------------------------------------------------------------------------------------------------------------------------------------

**HIV monitoring, loss, return, interruption of art and restarting**

Risk of loss to follow-up per 3 mths among those not on ART (rate_lost): 0.05

Probability of simultaneously being lost to follow-up amongst those stopping ART (prob_lost_art): 3/11

Probability (per 3 mths) of return to care for person lost (if no WHO 4 condition present – value is 1 if present) (rate_return): 0.05

Basic probability of restart of ART in those remaining under care who have stopped/interrupted ART (this is also influenced by presence of WHO 3 or 4 conditions) (rate_restart): 0.2

Probability of ART initiation per 3 months after eligibility fulfilled, if visting clinic: 0.5

-----------------------------------------------------------------------------------------------------------------------------------------------

**ART**

ART introduction date: 2003

Probability of switching to second line treatment, given first line failure (by whatever definition is being used) (pr_switch_line): 0.03 (changes to 0.3 after t0, 2017)

Pattern of adherence:

5% of people: average long term adherence = 0.49 ; period to period variability in adherence = 0.20

10% of people: average long term adherence = 0.79 ; period to period variability in adherence = 0.20

27% of people: average long term adherence = 0.90 ; period to period variability in adherence = 0.06

38% of people: average long term adherence = 0.90 ; period to period variability in adherence = 0.05

20% of people: average long term adherence = 0.95 ; period to period variability in adherence = 0.05

Reduction in adherence resulting from presence of TB or a WHO 4 condition (red_adh_tb_adc): 0.1

Average reduction in adherence resuting from current toxicity (the actual reduction varies by inidvidual person) (red_adh_tox_pop): 0.05

Additional "effective" adherence for people on NNRTI regimens due to longer half life (add_eff_adh_nnrti): 0.1

Average change in adherence on second line (degree of change varies by indvidual – note this can be a positive or negative change) (altered_adh_sec_line_pop) = 0.05

Proportion of people for whom a measured viral load > 1000 leads to an improvement in adherence (for the first time such a measurement is made only) (adh_effect_of_meas_alert): 0.7

Extent to which the CD4 change is more favourable on a virologically failing BPI-regimen compared with an NNRTI-regimen (poorer_cd4_rise_on_failing_nnrti): -6

Standard deviation for intra-subject variation in CD4 count (sd_cd4): 1.2

Standard deviation for the measurement error in CD4 count (sd_measured_cd4): 1.7

Base probability of interrupting ART per 3 mths (actual probability also depends on time on continuous ART, presence of current toxicity and average adherence – see model details) (rate_int_choice): 0.02

Probability of drug stock out, and hence ART initerrupted (prob_supply_interrupted): 0.01

Probability that drug supply resumed during stock-out (prob_supply_resumed): 0.8

Probability of NNRTI-resistance emerging in women taking SD nevirapine for PMTCT: 0.35

Probability of NNRTI-resistance emerging in women taking nevirapine plus at least one other antiretroviral for PMTCT: 0.045

Fold difference in risk of mutations arising, for given number of active drugs, viral load and current adherence level, compared with base risk (see model details) (fold_change_mut_risk): 1

Similarly, specifically for thymidine analogue mutations: (fold_change_tams_risk): 1

Similarly, specifically for Q151M cross nucleoside resistance mutation: (fold_change_151_risk): 1

Standard deviation representing inter-patient variation in rate of CD4 rise - when CD4 is rising (sd_patient_cd4_rise_art): 0.2

Risk of NNRTI-resistance emergence due to stopping an NNRTI regimen (due to the tail in presence of drug meaning effective monotherapy): 0.03

Fraction of people who stop ART (and are still visiting the clinic) for whom the clinic is not aware of the interruption and is hence treating the patient as if they were on ART (and hence may switch to the next line having wrongly classified them as virologically failing): (clinic_not_aw_int_frac): 0.6

-----------------------------------------------------------------------------------------------------------------------------------------------

**Costs**

All costs are in $1000 per 3 month period.

--------------------------------------------------------------------------------------------------------------------------------------------------

Drug costs* below increased by 20%** for supply chain etc – costs are annual unless stated

zidovudine: 0.070

tenofovir: 0.048

ddI: 0.100

lamivudine: 0.021

stavudine: 0.024

nevirapine: 0.028

efavirenz: 0.039

lopinavir/r: 0.268

atazanavir/r: 0.219

Mean cost of treatment of a WHO 4 condition over 3 months (cost is incurred for 3 months): 0.200^+^

Mean cost of treatment of a WHO 3 condition over 3 months (cost is incurred for 3 months): 0.020^+^

Mean cost of treatment of TB per 3 months (cost is incurred for 6 months): 0.050^+^

Cotrimoxazole annual cost: 0.005 ^+^

CD4 count measurement: 0.010 *** ^+^

Viral load measurement: 0.045 *** ^+^

Clinic visit cost: 0.020 ^+^

Resistance test cost: 0.300 ^+++^

HIV test (including personnel costs): 0.010 **

--------------------------------------------------------------------------------------------------------------------------------------------------

* Untangling the web of antiretroviral price reductions. 16th Edition – July 2013. [www.msfaccess.org](http://www.msfaccess.org/).

** Eaton J et al. How should HIV programmes respond to evidence for the benefits of earlier treatment initiation ? A combined analysis of 12 mathematical models. Lancet Global Health 2013.

***Keebler D, Revill P, et al. How Should HIV Programmes Monitor Adults on ART? A Combined Analysis of Three Mathematical Models. Lancet Global Health 2013.

+ Specific data not available on average unit costs of treating WHO stage 3 and 4 conditions and per clinic visit costs - costs used are informed by evidence synthesis from studies that cost according to current CD4 count of those in pre-ART care, cost of ART initiation, which also include costs of CD4 tests (Eaton J et al.)

^+++^ estimate of potential lowest feasible cost with centralised testing.

**Utilities***

Values are 1 except for the following:

Any drug toxicity in current 3 month period: 0.95

Any WHO 3 condition (except TB) in current 3 month period: 0.78

Current TB in current 3 month period: 0.60

Any WHO 4 condition in current 3 month period: 0.46

* Salomon JA, Vos T, Hogan DR, et al. Common values in assessing health outcomes from disease and injury: disability weights measurement study for the Global Burden of Disease Study 2010. Lancet 2012; 380: 2129–43. (20)
